# Structural Relationships Among Mental Boundaries, Childhood Imaginary Companions, Creative Experiences, and Entity Encounters

**DOI:** 10.1177/00332941221123235

**Published:** 2022-08-22

**Authors:** Kenneth Drinkwater, Neil Dagnall, James Houran, Andrew Denovan, Ciarán O’Keeffe

**Affiliations:** 5289 Manchester Metropolitan University, Manchester, UK; Laboratory for Statistics and Computation, 586197 ISLA—Instituto Politécnico de Gestão e Tecnologia; and Integrated Knowledge Systems, Inc., Dallas, TX, USA; 4013 University of Huddersfield, Huddersfield, UK; Buckinghamshire New University, High Wycombe, UK

**Keywords:** Encounter experiences, imaginary companions, transliminality, creativity, boundary functioning

## Abstract

This study investigated relationships between thin mental boundary functioning, creativity, imaginary companions (ICs), and anomalous ‘(entity) encounter experiences.’ A convenience sample of 389 respondents completed the Revised Transliminality Scale, Oxford-Liverpool Inventory of Feelings and Experiences, Creative Experiences Questionnaire, Survey of Strange Events, and a measure of Childhood Imaginary Companions. Competing testing with path analysis found that the best-fitting model was consistent with the causal chain of ‘Thin Boundaries (transliminality and schizotypy) → Creative Experiences → ICs → (Entity) Encounter Experiences.’ These results suggest that deep-types of ICs (i.e., showing apparent independent agency) are perhaps most accurately characterized as syncretic cognitions versus hallucination-like experiences. The authors examine these findings relative to study limitations, as well as discussing the need for future research that approaches ICs as a special mental state that can facilitate allied altered-anomalous experiences. In this context, this study furthered understanding of relationships between conscious states related to mental boundaries, childhood imaginary companions, creative experiences, and entity encounters.

Imaginary companions (ICs) are defined as self-generated, fantasy characters with whom children converse and interact directly for an extended period. Hence, ICs can develop into an enduring feature of childhood, lasting for several months or years (Taylor et al., 2004). Despite apparently having no objective basis, ICs possess an air of reality for experiencers (Svendsen, 1934) that can include detailed physical characteristics and personalities (Armah & Landers-Potts, 2021; Fernyhough et al., 2019; Giménez-Dasi, Pons, & Bender, 2014; Taylor, 1999). Moreover, the definition of ICs has been extended to include personified objects, which involve make-believe beings embodied within a toy or other object (Aguiar et al., 2017; Armah & Landers-Potts, 2021; Gleason, 2005). Related research also supports parallels with pretend identities (Moriguchi & Todo, 2018; Taylor, Shawber, & Mannering, 2009), as children who experience these phenomena share abilities and personality characteristics that distinguish them from individuals without imaginary constructions (Gleason et al., 2003; Taylor, 1999).

Experiences of childhood ICs are relatively common, but methodological and conceptual variations prevent precise incidence rates Accordingly, previous studies have reported figures as high as 65% (Armah & Landers-Potts, 2021), though most typically between 20% and 35% (Giménez-Dasi et al., 2014). A further feature is that IC character formation ranges from *shallow* (i.e., basically copies of the children who invented them) to *deep* (i.e., characters that seem alive and with independent wills) (e.g., Fernyhough et al., 2019; Hoff, 2005). Further to these deep forms, some of the children in Hoff (2005) reported ICs that seemed so much ‘alive’ that they forgot these ‘friends’ were imaginary. Research on accounts of deep ICs suggest that these instances might be best conceptualized as ‘(entity) encounter experiences’ (EEs) (Little et al., 2021).

By way of explanation, the empirical literature indicates that (a) many persons report EEs, i.e., perceived interactions with anomalous beings or sentient forces (Evans, 1987; Kumar & Pekala, 2001), (b) EEs comprise a reliable set of ‘subjective and objective’ (*S/O*) events in an experient’s environment (Houran, Lange et al., 2019: Houran, Laythe et al., 2019), (c) experients have an ‘encounter-prone’ profile rooted in transliminality (or ‘thin’ mental boundaries) (Laythe et al., 2018) that ostensibly facilitates recurrent and diverse *S/O* perceptions typical of ‘Haunted People Syndrome’ (Lange et al., 2020; Laythe, Houran, Dagnall, & Drinkwater, 2021; O’Keeffe et al., 2019), and (d) the particular attribution or interpretation of EEs often follows from the sociocultural perspective in which they occur (Houran, 2000). Accordingly, Little and colleagues (2021; cf. Laythe, Houran, & Little, 2021) proposed that deep ICs could be ‘disguised or overlooked’ EEs that represent a hybrid between ‘spontaneous’ and ‘induced’ types of altered-anomalous experiences.

In this context, some children (and adults) might possess the ability to generate EEs in different sensory modes on demand, in naturalistic settings, and during apparent normal waking states. This view of ICs, and especially as related to their deep forms, suggests these are hallucination-like experiences with potentially adaptive value for the development of social cognition (Davis et al., 2011), creativity (Hoff, 2005), or inner speech, i.e., internal voices that combine conscious thoughts and unconscious beliefs and biases to help interpret and process questions, ideas, or experiences (Fernyhough et al., 2019). Indeed, there is a burgeoning literature on creativity in relation to hallucinatory, metachoric, and syncretic experiences (e.g., D’Anselmo et al., 2020; Fink et al., 2014; Green, 1990; Wilson & Barber, 1981) — all of which positively correlate with thin mental boundary functioning (Evans et al., 2019).

Research linking thin boundary functioning to ICs has also implicated schizotypal tendencies (Jones et al., 2015), which are a set of personality traits or characteristics that represent a latent personality construct or liability to develop schizophrenia (Lenzenweger, 2010). This finding is not inconsistent with a transliminal model of ICs, which views transliminality and schizotypy as overlapping constructs (Thalbourne et al., 2005). Schizotypal aspects to certain ICs imply that these experiences do not always have efficacious outcomes. Specifically, that deeper forms of IC could reflect cognitive bandwagon effects such as manifestations of depersonalization, derealization, or dissociated identity (for a discussion, see Lange et al., submitted)

Based on preceding research, we hypothesized relationships between thin mental boundary functioning, creativity, imaginary companions (ICs), and anomalous ‘(entity) encounter experiences.’ Specifically, we proposed a process model, whereby an encounter-prone profile rooted in transliminality and schizotypal tendences promotes creativity, which facilitates the development of ICs, and eventually a broader array of EEs in the form of *S/O* type haunt perceptions. This paper tested this premise using path analysis. This technique, a subset of structural equation modeling, is a statistical method for investigating direct and indirect relationships among a set of exogenous and endogenous variables (Bollen & Pearl, 2013; Wuensch, 2016; Zhang & Wang, 2017). It is basically a generalization of regression and mediation analysis where multiple input, mediators, and output can be used. The pattern of relationships among variables is described by a path diagram, which is a type of directed graph. Variables are linked by straight arrows that indicate the directions of the causal relationships between them. This modeling approach has been used in several previous studies of psychosocial influences on anomalous beliefs and experiences (see Cicero et al., 2021; Lange & Houran, 1999; Lawrence et al., 1995).

## Method

### Respondents

Data comprised a convenience sample of 389 respondents. Within this, 158 participants (41%, *M*_age_ = 39.07, *SD* = 14.20) reported an imaginary companion. Thirty-one were male (*M*_age_ = 43.03, *SD* = 16.08, 18–75 years), 119 female (*M*_age_ = 38.32, *SD* = 13.66, 18–75 years), and eight did not specify (*M*_age_ = 34.75, *SD* = 11.06, 22–55 years). Two hundred and thirty-one (59%) (*M*_age_ = 41.32, *SD* = 13.95, 18–83 years) did not report an imaginary companion. Sixty-eight were male (*M*_age_ = 45.13, *SD* = 13.73, 19–74 years), 158 female (*M*_age_ = 39.82, *SD* = 13.74, 18–83), and five did not specify (*M*_age_ = 37.00, *SD* = 16.17, 24–64). Respondents were recruited using a snowball sampling approach across different UK universities. Only participants reporting an imaginary companion were used in this study.

### Measures

#### Phenomenology of Imaginary Companions

Following a literature search of measures used to assess the presence and characteristics of ICs, the authors adapted several sourced instruments for the present study. To determine whether respondents had an IC during childhood they were issued with a definition (“Pretend friends are ones that are make-believe, that you pretend are real”; Taylor et al., 1993) accompanied by a response item (“Did you ever have an imaginary friend growing up?”; Auton et al., 2003). If respondents answered “No” they skipped the subsequent IC-related items and progressed to the other study sections.

If respondents answered “yes” a further set of items asked for specific information to determine whether ICs were best categorized as *shallow* (i.e., basically copies of the children who invented them) or *deep* (i.e., characters that seemed alive and with independent wills) (Fernyhough et al., 2019; Hoff, 2005). An example of ‘shallow IC’ is a respondent who endorses the statement “My imaginary friend played with me when I was lonely,” whereas a sample ‘deep IC’ item is the endorsement of the statement “My imaginary friend tried to boss me around.” Thus, we asked about the IC’s name, substance (i.e., toy or completely pretend), gender, age, physical appearance (i.e., what did they look like), and their (dis)likes (see Taylor et al., 1993). A further item asked about the number of imaginary friends. If respondents indicated that they had multiple companions (Silberg, 2013), for the remaining items they were told to focus on their most ‘significant’ imaginary friend. Questions, requiring “Yes/No” responses, enquired about where the IC lived and slept, how old the respondent was when they first met their IC, how old they were when the friend disappeared, and dislikes about their imaginary companion. The final set of “Yes/No” items, derived from Hoff (2005), Majors and Baines (2017), and Silberg (2013), asked respondents about the types of interactions they had with their imaginary friends, i.e., the types of interaction(s) and purpose(s), play activities and whether siblings also had ICs. For example, “Did you and your real friends play with the pretend friend?”, “With your pretend friend, did you interact with real objects?”, and “Did you visit real places together?”

#### The Oxford-Liverpool Inventory of Feelings and Experiences short version (sO-Life)

This is an abridged version of the original 104-item scale that assesses schizotypal personality traits in non-clinical samples (Mason et al., 1995). The measure comprises 43-items, indexing four sub-scales: Unusual Experiences (UnExp), Cognitive Disorganization (CogDis), Introvertive Anhedonia (IntAn), and Impulsive Nonconformity (ImpNon) (Mason et al., 2005).

UnExps measures positive schizotypy (magical thinking, perceptual aberrations, and hallucinations) using 12-items (e.g., “Are your thoughts sometimes so strong that you can almost hear them?”). CogDis (11-items) assesses the presence of thought disorder and other disorganized aspects of psychosis. Particularly, items (e.g., “Do you frequently have difficulty in starting to do things?”) reference poor attention/concentration, flawed decision-making, and social anxiety. IntAn (10-items) references negative schizotypy (schizoid temperament). Explicitly, items (e.g., “Are there very few things that you have ever enjoyed doing?”) capture lack of enjoyment from social and physical sources of pleasure, and avoidance of intimacy. ImpNon indexes lack of self-control (impulsive, anti-social, and eccentric behaviour) using 10-items (e.g., “Do you at times have an urge to do something harmful or shocking?”). Items appear as statements and respondents indicate agreement on a dichotomous YES/NO scale. In addition to sub-scale scores, summation of items produces an overall measure of schizotypy. The sO-Life is a widely used measure that possesses recognised psychometric qualities. Specifically, reliability (internal and test–retest reliability) and validity (Fonseca-Pedrero et al., 2015; Mason & Claridge, 2006).

*Revised Transliminality Scale* (RTS: Lange, Thalbourne, Houran, & Storm, 2000) is a 17-item, T/F, Rasch-scaled measure of “hypersensitivity to psychological material originating in (a) the unconscious, and/or (b) the external environment” (Thalbourne & Maltby, 2008, p. 1618). Thus, this perceptual-personality variable parallels Hartmann’s (1991) mental boundary construct and the notion of sensory processing sensitivity (Aron & Aron, 1997). An example item is “At times I perform certain little rituals to ward off negative influences”. The Rasch reliability is .82, and RTS scores (*M* = 25, *SD* = 5) consistently predict different syncretic cognitions and lower psychophysiological thresholds (for reviews, see Evans et al., 2019; Lange et al., 2019).

The *Creative Experiences Questionnaire* (CEQ: Merckelbach et al., 2001) contains 25 “yes/no” statements (e.g., “As a child, I often felt lonely”) derived from case descriptions indexing fantasy proneness (Wilson & Barber, 1981). Scores thus range from 0–25, with higher scores indicating greater fantasy proneness. The CEQ is an established, psychometrically robust measure from a Classical Test Theory perspective (i.e., possesses good internal and test-retest reliability) (Merckelbach et al., 2001).

*Survey of Strange Events* (SSE: Houran, Laythe, et al., 2019). This is a 32-item, ‘true/false’ Rasch scaled measure of the overall intensity (or perceptual depth) of a ‘ghostly-entity encounter’ narrative via a weighted checklist of base events (subjective/psychological and objective/physical) inherent to these episodes. Note that a ‘logit’ denotes the locations of items within the Rasch hierarchy, with higher logit values indicating higher positions (or greater difficulty) on the scale (for a discussion, see Bond & Fox, 2013). An example item is “I had a negative feeling for no obvious reason, like anger, sadness, panic, or danger”. We refer readers to our previous papers for details on the development of this instrument (Houran, Lange, et al., 2019; 2019b).

Rasch scaled scores range from 22.3 (= raw score of 0) to 90.9 (= raw score of 32), with a mean of 50, *SD* = 10, and a Rasch reliability = 0.87. Higher scores correspond to a greater number and intensity of anomalies that define a percipient’s experience. Furthermore, supporting the SSE’s content and predictive validities, Houran, Laythe, et al. (2019) found that the phenomenology of “spontaneous” accounts (i.e., ostensibly “sincere and unprimed”) differed significantly from “control” narratives from “primed conditions, fantasy scenarios, or deliberate fabrication.” Follow-up studies with the SSE also support its value for content or thematic analyses of qualitative reports (Lange et al., 2020; Laythe et al., 2021; O’Keeffe et al., 2019).

### Procedure

All respondents completed the measures online. The study was accessed via a web link hosted by the Qualtrics survey tool. Prior to opening study measures respondents received a detailed brief. This outlined the nature of the study and ethical procedures. If respondents consented to participate, they registered informed consent and progressed to the measurement items and scales. Procedural instructions told respondents to read questions carefully; progress through the survey systematically, at their own pace; complete all questions; and provide honest and open answers. Demographic information appeared first (i.e., preferred gender, age, and general location), followed by the sections on imaginary companions and The Survey of Strange Events was always completed last. As indicated earlier, the Survey of Strange Events (SSE) is a measure of the overall intensity of a ‘ghostly-entity encounter’ narrative with items, therefore, referring to ghostly or apparition-like figures. Additionally, there are some items that refer to “angels”, “demons”, “elves”, “fairies”. Given the potential for priming respondents to initially conceptualise their own IC as a ghostly or mythical figure by having the SSE earlier in the questionnaire battery, we ensured SSE was always completed last. The order of the sO-Life, Revised Transliminality Scale, and Creative Experiences Questionnaire rotated across respondents to counter order effects.

This study used a cross section approach, where data were collected at one time point. A criticism of this approach is that it can result in common method variance (CMV) (Spector, 2019). This arises when scales affect responses, producing bias. To prevent CMV, we applied a series of procedural counter-measures (Krishnaveni & Deepa, 2013). Firstly, to reduce social desirability and evaluation apprehension, instructions emphasized that honesty was important and that there were no correct responses. Secondly, to ensure psychological distance between constructs the study brief followed recommended guidelines by stating that all measurement items and scales were independent (Podsakoff et al., 2003). Respondents were debriefed on the survey’s completion.

## Results

### Descriptive Statistics

Descriptive statistics and zero-order correlations appear in Table 1. Data screening for univariate normality revealed that skewness was within the recommended range of −2.0 to +2.0 (Byrne, 2016). Further, Mardia’s (1970) test of multivariate normality revealed no issues, as the test coefficient (2.46) was lower than the cutoff of 3. All study variables showed moderate to strong positive correlations (see Table 1).

**Table 1.** Means, Standard Deviations and Correlations for all Study Variables (*N* = 421).

Variable	*M*	*SD*	Skew	1	2	3	4	5
1. sO-Life	19.31	7.48	0.43		0.58**	0.63**	0.39**	0.35**
2. RTS	26.20	4.49	0.22			0.70**	0.38**	0.50**
3. CEQ	12.51	4.88	−0.07				0.34**	0.42**
4. IC	51.02	9.64	0.25					0.43**
5. SSE	44.97	14.26	−1.03					

*Note.* sO-Life = Oxford-Liverpool inventory of feelings and experiences short version; RTS = revised transliminality scale; CEQ = creative experiences questionnaire; IC = imaginary companion scale; SSE = survey of strange events. *p < 0.05; **p < 0.001.

### Path Analyses

**Figure 1.**
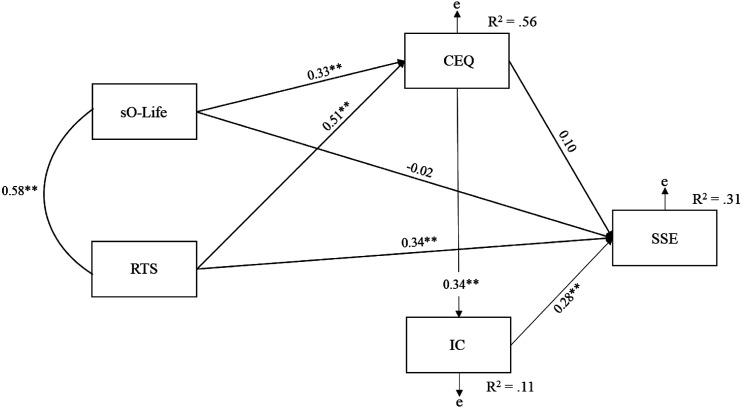
Initial sequential path model depicting relationships between schizotypy, transliminality, creative experiences, imaginary companions, and broader (entity) encounter experiences. *Note*: Observed variables are depicted by rectangles; error is represented by ‘e’. sO-Life = Oxford-Liverpool inventory of feelings and experiences short version; RTS = revised transliminality scale; CEQ = creative experiences questionnaire; IC = imaginary companion scale; SSE = survey of strange events. *p < 0.05; **p < 0.001.

We assessed our hypothesised mediation model using the *AMOS25* software (see Figure 1). Absolute and comparative fit indices evaluated data-model fit. Absolute fit indices included the Standardised Root-Mean-Square Residual (SRMR), which examines the mean absolute correlation residual. Smaller values specify better model fit. The Tucker Lewis Index (TLI) and Comparative Fit Index (CFI) examine the discrepancy between observed data and the hypothesized model. Larger values signify better fit (and thereby less discrepancy). According to Browne and Cudeck (1993), an acceptable model requires SRMR < 0.08, TLI > 0.90 and CFI > 0.90. Model comparison included consultation of Akaike’s Information Criterion (AIC), with lower values representing superior fit.

**Figure 2.**
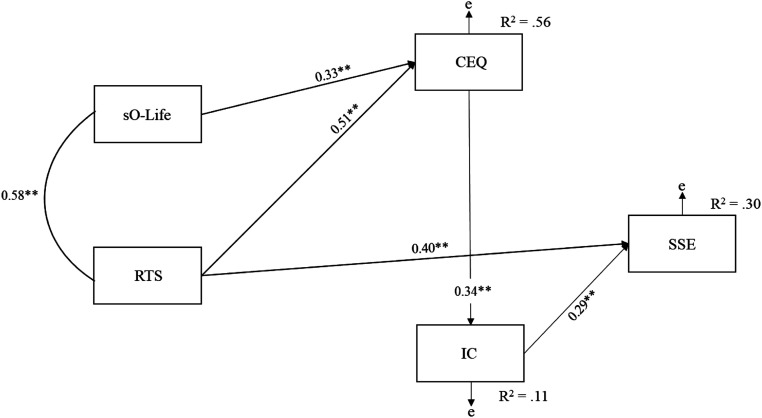
Revised sequential path model depicting relationships between schizotypy, transliminality, creative experiences, imaginary companions, and broader (entity) encounter experiences. *Note*: Observed variables are depicted by rectangles; error is represented by ‘e’. sO-Life = Oxford-Liverpool inventory of feelings and experiences short version; RTS = revised transliminality scale; CEQ = creative experiences questionnaire; IC = imaginary companion scale; SSE = survey of strange events. *p < 0.05; **p < 0.001.

Analysis found acceptable fit on all indices, except the TLI, χ^2^ (2, *N* = 156) = 13.09, *p* = 0.001, TLI = 0.80, CFI = 0.96, SRMR = 0.06. Consideration of path estimates revealed the presence of non-significant paths between sO-Life and SSE (0.02), and CEQ and SSE (0.10). Therefore, analysis investigated a model with these paths fixed to zero (Figure 2). This demonstrated good fit on all indices, χ^2^ (4, *N* = 156) = 14.15, *p* = 0.007, TLI = 0.91, CFI = 0.96, SRMR = 0.06. Additionally, a lower AIC existed compared with the initial solution (49.09 vs. 46.15).

The path relationships showed significant and positive direct effects of RTS (0.51, *p* < 0.001 [0.41, 0.61]) and sO-Life (0.33, *p* = 0.002 [0.20, 0.45]) on CEQ, RTS on SSE (0.40, *p* = 0.002 [0.27, 0.52]), CEQ on IC (0.34, *p* = 0.002 [0.21, 0.45]), and IC on SSE (0.29, *p* = 0.002 [0.13, 0.43]). Analysis computed indirect effects of sO-Life and RTS on SSE through the sequential paths of CEQ and IC, drawing on 1000 bias-corrected bootstrap resamples. sO-Life had a significant indirect effect on SSE, 0.06, *p* < 0.001 [0.02, 0.13]. In addition, RTS had a significant standardised indirect effect on SSE, 0.16, *p* < 0.001 [0.06, 0.35].

## Discussion

Interestingly, 41% of our respondents reported a childhood IC. This figure seems high, but it concurs with previous research suggesting an incidence rate between 20% and 35% (Giménez-Dasi et al., 2014). Though ICs are clearly common experiences, their phenomenology and potential nature and functions have remained enigmatic. The present results are arguably the first to show empirically, however, that thin (permeable) mental boundaries (operationalized via Transliminality and Schizotypy) encourage Creative Experiences, which subsequently facilitate the development of ICs. These results correspondingly support the hypothesis that ‘deep’ ICs are associated with the manifestation of various other Encounter Experiences (EEs). Transliminality also predicted a broader array of EEs, as measured by the SSE. The best-fitting path model in Figure 2 affirmed prior speculations of a direct link between ICs and EEs, as well as the idea that boundary functioning directly or indirectly facilitates IC phenomena (see Laythe et al., 2018, 2021b; Little et al., 2021; Ventola et al., 2019). Broadly, the finding that Transliminality and Schizotypy —two constructs related to altered and productive cognitive-perceptual experiences— predicted adult-reporting of childhood ICs agrees with Firth et al.’s (2015) finding that adults having had childhood ICs rated themselves as more imaginative and scored higher on an objective measure of imaginative capacity (i.e., scene construction task).

Overall, the present results suggest that Transliminality might be a common factor that can account both for dysfunctional and non-dysfunctional childhood ICs (Taylor, 1999). The observation that thin boundary functioning enables ICs indicates that these experiences are likely more than just ‘hallucination-like experiences’ (Fernyhough et al., 2019). Transliminality is hypothesized to reflect enhanced interconnectedness between brain hemispheres, as well as among frontal cortical loops, temporal-limbic structures and primary or secondary sensory areas or sensory association cortices (for an overview, see Evans et al., 2019). From this perspective, deep ICs could represent expressions of syncretic cognitions. This refers to the dedifferentiation (or fusion) of perceptual qualities in subjective experience, such as *physiognomic perception* (i.e., fusion of perception and feeling); *synesthesia* (i.e., fusion of sensory modalities), and *eidetic imagery* (i.e., fusion of imagery and perception, i.e., structural eidetic imagery) occurs (see e.g., Evans et al., 2019; Lange et al., 2019). This idea might help to explain how IC experiences comprise different sensory modalities (Fernyhough et al., 2019).

Of course, direct replications with real-time data from children (and adults who report ICs) are needed to corroborate both our preliminary process model and its implications. This should certainly include larger and more diverse samples to ensure that the findings remain robust regardless of demographic differences. Additionally, although previous validation studies of the sO-Life have firmly supported the four-factor model, more recently a three-factor (without the Impulsive Nonconformity sub-scale) has been demonstrated. Fonseca-Pedrero et al. (2015) have proposed that the inclusion of this latter dimension is dependent on theoretical arguments and research goals. Additionally, due to the possibility of measurement invariance not holding, they further state that “comparability between different groups only makes sense if it can be guaranteed that participants interpret and understand the items of the latent construct in a similar manner,” (Fonseca-Pedrero et al., 2015, p. 342). This latter point therefore argues for a more in-depth examination of the sO-Life factors (sub-scales) with the other measures. This could include the use of the original 104-item questionnaire (Mason et al., 1995) and applying more advanced psychometric analyses grounded in Modern Test Theory (Lange, 2017). As noted previously, the SSE also value for content or thematic analyses of qualitative accounts of EEs (Lange et al., 2020; O’Keeffe et al., 2019; Laythe, Houran, & Little, 2021). There is scope, then, of collating and analyzing more free-flowing IC narratives using the SSE but also exploring the phenomenology of “spontaneous” versus “control” accounts and the relationship with Transliminality, CEQ, etc. This type of qualitative examination would potentially facilitate real-time data collection on ICs from children (or adults).

Notwithstanding the preliminary nature of this study, the present results suggest, clinically speaking, that children with deep ICs are expected to perceive a wide array of anomalous experiences that transcends a ‘solitary’ imaginary companion. These additional and repeated occurrences can be construed as a series of mini-EEs (Houran, Laythe, et al., 2019) that might cause the child experient to feel in the company of, or even ‘haunted’ by, what seem to autonomous beings or agencies (Laythe, Houran, & Little, 2021). Thus, as illustrated by Jones et al.(2015), clinicians might easily mistake reports of such anomalous experiences as positive symptomatology per a biomedical model. We would caution against making such automatic assumptions. More research is clearly needed to differentiate what might be manifestations of heightened (but non-pathological) levels of creativity and transliminality from certifiable issues of mental illness or impairment (see Johnson & Friedman, 2008).

Extending the current paper, ensuing research could examine the role that reality testing plays in validation of imaginary companions. This is necessary because proneness to reality testing correlates positively with Schizotypy (Dagnall et al., 2017; Denovan et al., 2020) and Transliminality (Dagnall et al., 2010). These relationships are in the medium to large range as identified by Gignac and Szodorai (2016) and therefore of potential conceptual importance. Additionally, reality testing is a factor associated with both imagination and experience of fantasy friends. For example, following elicitation of a description of a monster, Bouldin and Pratt (2001) observed that children with (vs. without) imaginary companions were more responsive to a monster-shaped silhouette within a tent (i.e., more frequently reported that they had seen a monster).

These findings indicate that children with ICs more readily embrace the possibility that unreal representations reflect reality. Acknowledging this evidence, the Inventory of Personality Organization Reality Testing Subscale (IPO-RT) (Lenzenweger et al., 2001) merits inclusion. The IPO-RT assesses the capacity to differentiate self from non-self and intrapsychic from external stimuli (Kernberg, 1996). Explicitly, the scale measures overreliance on intrapsychic data. This manifests as the tendency to draw conclusions about the world based on internally generated rather than externally available data. The IPO-RT is particularly useful in the context of ICs because in addition to overall scores, the scale possesses subscales: Hallucinations (auditory and visual), Delusional Thinking (beliefs contrary to reality), Social Deficits (difficulties reading social cues), and sensory/perceptual ‘confusion’ (inability to understand) (Dagnall et al., 2018). Consideration of reality testing deficits at the factorial level may indicate, which facets are most strongly related to ICs.

Additionally, future research could extend the present paper to consider the extent to which experience of ICs endures from childhood into adulthood. This is important because consideration of ICs is typically limited to formative development years (Fernyhough et al., 2019). For some individuals, ICs sustain into adulthood where they potentially serve important psychological functions. This would potentially indicate whether adulthood ICs are merely extensions of childhood experiences, change over time, or are quantitively different. That is the, while they are a product of a creative-immersive psychological style, they possess different features and characteristics.

The present study was cross-sectional and based on relationships. Therefore, it is not possible to establish causation. Thus, it is unclear whether higher levels of Transliminality and Schizotypy produce ICs, or a consequence of ICs is heightened scores on these constructs. Furthermore, scholars report that cross-sectional data sometimes provide biased estimates (Maxwell & Cole, 2007). It is thus important for future research to carry out longitudinal assessments to validate fully a causal relationship from schizotypy/transliminality to imaginary companions and the perception of anomalous experiences or events that characterize other types of entity encounter experiences. In relation to this limitation, it is noteworthy that there are theoretical arguments supporting the more primitive status of schizotypal traits. Particularly, schizotypy is a trait-like construct with a notable genetic component (see e.g., Ericson et al., 2011). It might also be valuable to examine the relationships modeled here using experimental designs such as with mirror-gazing protocols (Caputo et al., 2021), as well as the potential role of other cognitive or perceptual variables including trait absorption, Big Five or Six personality traits, daydreaming (and possibly maladaptive daydreaming). idiopathic environmental intolerance, or mental toughness. We also recommend exploring the influences of stress or trauma, as suggested by the research linking dis-ease states to encounter experiences (for a discussion, see Laythe, Houran, Dagnall, & Drinkwater, 2021).

A further limitation is that data generation relied upon retrospective recall of childhood experiences. This can prove a flawed process due to internal (i.e., memory fallibility) and external factors (i.e., the reaction of significant others to the fantasy friend) (Brewin et al., 1993; Dagnall et al., 2008). This could be less of a concern in the present study, since the key determining factor was whether respondents reported having had a childhood IC. Thus, the additional questions acted as a veracity check. Noting the issues associated with retrospective recall, subsequent studies, especially those considering the perceived properties of ICs, could seek collaboration from other sources (e.g., parents and siblings) or structured methods such as interviews rather than merely relying on self-report measures. To be sure, the structure, phenomenology, and functions of ICs in childhood and adulthood is ripe area for future research that can effectively draw on multidisciplinary approaches used in the broad domain of consciousness studies.
